# The Impact of Multimodal Large Language Models on Health Care’s Future

**DOI:** 10.2196/52865

**Published:** 2023-11-02

**Authors:** Bertalan Meskó

**Affiliations:** 1 The Medical Futurist Intitute Budapest, XI. Hungary

**Keywords:** artificial intelligence, ChatGPT, digital health, future, GPT-4, Generative Pre-Trained Transformer, large language models, multimodality, technology, AI, LLM

## Abstract

When large language models (LLMs) were introduced to the public at large in late 2022 with ChatGPT (OpenAI), the interest was unprecedented, with more than 1 billion unique users within 90 days. Until the introduction of Generative Pre-trained Transformer 4 (GPT-4) in March 2023, these LLMs only contained a single mode—text. As medicine is a multimodal discipline, the potential future versions of LLMs that can handle multimodality—meaning that they could interpret and generate not only text but also images, videos, sound, and even comprehensive documents—can be conceptualized as a significant evolution in the field of artificial intelligence (AI). This paper zooms in on the new potential of generative AI, a new form of AI that also includes tools such as LLMs, through the achievement of multimodal inputs of text, images, and speech on health care’s future. We present several futuristic scenarios to illustrate the potential path forward as multimodal LLMs (M-LLMs) could represent the gateway between health care professionals and using AI for medical purposes. It is important to point out, though, that despite the unprecedented potential of generative AI in the form of M-LLMs, the human touch in medicine remains irreplaceable. AI should be seen as a tool that can augment health care professionals rather than replace them. It is also important to consider the human aspects of health care—empathy, understanding, and the doctor-patient relationship—when deploying AI.

## The Current State of Large Language Models in Health Care and Medicine

When large language models (LLMs) were introduced to the public at large in late 2022 with ChatGPT (OpenAI), the interest by the general public was unprecedented, with more than 1 billion unique users within 90 days [1]. LLMs are advanced machine learning algorithms designed to understand and generate human-like text, revolutionizing fields such as natural language processing, content creation, and information retrieval. ChatGPT, powered by the Generative Pre-trained Transformer (GPT) engine, has been the most popular representative of LLMs.

Until the introduction of GPT-4 in March 2023, these LLMs only contained a single mode—text. While using LLMs in the singular text mode, in itself, has major implications in medicine for documentation, the practice of health care is highly influenced by images and the interaction of clinicians with patients by voice during office visits and bedside rounds.

This paper zooms in on the new possibilities of generative artificial intelligence (AI), a new form of AI that also includes tools such as LLMs, through the achievement of multimodal inputs of text, images, and speech [2]. We present several futuristic patient scenarios to help illustrate the potential path forward, as multimodal LLMs (M-LLMs) could represent the gateway between health care professionals and using AI for medical purposes.

## Potential Future LLM Versions Could Handle a Range of Content Types

As medicine is a multimodal discipline, the potential future versions of LLMs that can handle multimodality—meaning that they could interpret and generate not only text but also images, videos, sound, and even comprehensive documents—can be conceptualized as a significant evolution in the field of AI. Such advancements would enable more holistic patient assessments, drawing from diverse data sources for accurate diagnoses and treatment recommendations. Furthermore, it would foster interdisciplinary collaboration by seamlessly integrating varied medical data, enhancing patient care, and facilitating groundbreaking research.

These hypothetical models could be termed M-LLMs and would be able to process, interpret, and generate information across multiple data types, greatly expanding the utility and applicability of LLMs. M-LLMs could provide interpretation of an image, the sentiment of a sound clip, and the context of a video and analyze the structure and content of a full document, in addition to editing text and simulating discussions.

In this paper, we focus on health care, medical, and research-related use cases. With text input, LLMs can already document patient encounters and medical histories, complete insurance forms and write insurance letters, solve case studies, or help develop treatment plans, among others.

By being able to analyze images, LLMs could also read handwritten notes and prescriptions, assess radiology images, analyze photos of skin lesions, identify diseases based on pathology slides of tissue samples, or detect retinopathy by analyzing images of the retina.

Being able to handle sound samples would open up new possibilities, such as looking for vocal biomarkers, performing cough analysis, analyzing heart and lung sounds for abnormalities, diagnosing sleep disorders such as sleep apnea, or translating spoken language into sign language for individuals with speech or hearing impairments.

With video content, there could be further advanced use cases, such as monitoring a patient’s progress in physical therapy or rehabilitation; aiding in surgical procedures by providing real-time insights; detecting neurological conditions such as Parkinson disease or epilepsy by recognizing patterns and symptoms such as tremors, seizures, or changes in gait; and accurately translating sign language to text or speech in real time.

Moreover, full document analyses could include literature review and research to aid clinical decision support; support learning for medical students by analyzing textbooks, papers, and teaching materials; facilitate public health by analyzing research papers and public health reports to track disease trends and predict outbreaks; or generate patient-friendly summaries or educational materials in patient education.

Multimedia Appendix 1 summarizes the 5 major categories of content types that future M-LLM versions could handle, with specific examples for each.

## Hypothetical Scenarios of M-LLMs

A few hypothetical scenarios might better demonstrate the potential of M-LLMs in the practice of medicine.

The first scenario is about remote diagnosis support. A rural health care provider is reviewing a complex case and turns to M-LLM for assistance, combining textual data with medical images.

Scenario 1: Remote patient diagnosis:

I have a patient with persistent abdominal pain. Here are the symptoms and a brief medical history. Please list the potential causes for differential diagnosis.

Doctor

Based on the described symptoms and medical history, it appears consistent with gallstones. Consider conducting an ultrasound for confirmation and exploring appropriate treatment options.

M-LLM

In the second scenario, a patient is using an integrated remote health platform powered by an M-LLM. After experiencing unusual symptoms following knee surgery, the patient uploads a photo of the swollen knee, shares recent heart rate data, and communicates concerns through text and voice interaction. The M-LLM analyzes these data, including the patient’s vocal biomarkers.

Scenario 2: Vocal biomarkers:

I had a knee surgery a few days ago. My knee is swollen, and my heart rate is up. Here’s a photo and data from my wearable. Should I be worried?

Patient

I’ve analyzed the photo of your knee and heart rate data. While the swelling seems within normal post-surgery reaction, the heart rate increase, and the anxiety I can detect in your voice merit attention. It might be helpful to discuss this with your physician. Would you like me to schedule an appointment? If so, please fill in this short form. [M-LLM] (Analyzing text, image, and data)

Yes, please. Can you find a time next week?

Patient

Certainly! I’ve found an opening with your physician next Tuesday at 10 am. If you would like me to book that for you, please fill in this short form.

M-LLM

Yes, that works. Thank you for the help!

Patient

You’re welcome! Your appointment will be confirmed soon. Continue to monitor your symptoms, and don’t hesitate to reach out if you need further assistance.

M-LLM

In the third scenario, a non–English-speaking patient connects with an English-speaking specialist through a telemedicine platform, aided by an M-LLM providing real-time translation.

Scenario 3: Breaking language barriers in telemedicine:

Can you describe the pain you’re experiencing?

Specialist

¿Puede describir el dolor que está sintiendo? [M-LLM] (Translating for a patient with cultural nuance awareness)

Siento un dolor agudo en el pecho.

Patient

I feel a sharp pain in the chest. [M-LLM] (Translating for a specialist with sensitivity to emotional undertones)

In the fourth scenario, a cardiac surgeon in training interacts with an M-LLM for a virtual reality surgical simulation, receiving real-time guidance and feedback.

Scenario 4: Surgical training:

I’m about to make the incision for a coronary artery bypass. Is my positioning correct?

Cardiac surgeon in training

Your angle is slightly off. Adjust your hand 15 degrees to the left, and ensure the scalpel is aligned with the marked line. Perfect, now proceed. [M-LLM] (Analyzing visual cues)

I’ve exposed the artery but notice some unexpected branching. How should I proceed?

Cardiac surgeon

The branching you see is a common anatomical variation. Use the clamp to gently isolate the branch in question, as I’m visually highlighting in your simulation. Continue as planned but be cautious around that area. M-LLM] (Analyzing real-time image)

Got it. I’m now suturing the graft. Any feedback?

Cardiac surgeon

Your suturing technique is good, but you can apply a bit more tension to ensure a secure fit. Well done! Would you like to review the procedure or move on to the next simulation? [M-LLM] (Evaluating virtual reality movements)

While such scenarios demonstrate the value and possibilities of M-LLMs analyzing a range of content types, such models also pose significant challenges.

Training these models would require extensive, diverse, and well-annotated data sets. Ethical and privacy concerns could be amplified, especially when handling sensitive data like medical images or personal documents. Finally, the computational requirements for training and deploying such models would likely be very high, potentially limiting their accessibility.

## The Power and Promise of Multimodality

### Overview

Currently, there are a few hundred AI-based, unimodal medical technologies with regulatory approvals worldwide [3]. According to a study on AI-based health care patents, thousands of similar medical technologies can be expected to reach the market in the coming years [4]. All of these technologies were trained on specific sets of data, meaning they were developed using distinct and often narrow data sets tailored to particular medical conditions or imaging modalities and can be used for precisely defined health care purposes.

While this is understandable, knowing how difficult and expensive the process of bringing AI-based medical technologies to the practice of medicine is, this setting represents a major barrier to the wide adoption of AI [5].

The advent of M-LLMs offers the promise of transcending these limitations, potentially introducing versatile and comprehensive solutions that could challenge the current state of specialized AI tools in medicine.

### Multimodal Analysis

Future versions could integrate and analyze various types of data, including text, images, audio, and video, allowing for a more comprehensive understanding of content. Consider a patient interacting with a telehealth platform powered by an M-LLM. The patient sends a message stating, “I’ve had a persistent cough for the last two weeks” (text input). Following this, they upload a photo of their inflamed throat (image input) and a short audio recording of their cough (audio input).

First, the M-LLM processes the text input, understanding the patient’s symptoms and the duration. It then analyzes the image, detecting signs of throat inflammation. Upon processing the audio input, it identifies the characteristics of the cough. Given these multimodal inputs, the M-LLM draws from its corpus of medical information to form a preliminary assessment. It might conclude the symptoms align with a respiratory infection, and then it proceeds to advise the patient to seek a consultation with a health care professional for a detailed examination and appropriate treatment. Furthermore, the model could generate a preliminary report summarizing the patient’s symptoms and observations made from the analysis of the image and audio inputs.

### An Upgraded Approach to Interoperability

Interoperability has been a major challenge in health care for decades [6]. By seamlessly analyzing and processing various data modalities, M-LLMs can bridge the gap between different medical software systems, such as electronic medical records, decision support tools, or radiology AI models. They have the ability to translate and align information into common formats, facilitating real-time collaboration and coordination across platforms. Consider a scenario where a radiology AI model’s insights are instantly integrated into a patient’s electronic medical record and matched with relevant guidelines in a decision support system, all orchestrated through an M-LLM. This level of integration paves the way for a more unified and responsive health care system where information flows smoothly, enhancing clinical decision-making, patient care, and overall efficiency.

### Breaking Down Language Barriers

M-LLMs could be capable of understanding and translating multiple languages, facilitating clear communication between health care providers and patients who speak different languages. This transcends simple translation, as context-aware understanding allows for the nuances of medical terminology to be accurately conveyed. Through M-LLMs, language barriers that once hindered health care accessibility and quality can be overcome, fostering a more inclusive and patient-centered care environment.

### Dealing With Advanced Scientific Data

Improved models might have a deeper understanding of scientific concepts and be able to analyze complex scientific data, such as research papers, experimental results, and simulations. Also, enhanced models could potentially process and analyze real-time data streams, enabling applications such as live event analysis, detecting emerging evidence in the literature, and dynamic data-driven insights in drug development.

### Sentiment and Contextual Analysis

M-LLMs might become more adept at understanding and analyzing human sentiment and emotion, enabling more accurate sentiment analysis for different types of content, including text, audio, and video. This could potentially be used to monitor mental health or patient recovery. Also, future models might better understand the context of a given situation, including the cultural, historical, and social nuances, leading to more accurate and context-aware analyses.

### Domain-Specific Analysis

Such models might specialize in analyzing specific domains, such as genomics, pharma, or radiology, providing domain-specific insights and recommendations. For example, M-LLMs might analyze genomic data to identify patterns that indicate a predisposition to certain diseases or optimize personalized treatment plans. This could greatly enhance precision medicine.

This could apply to technological directions too. They could analyze data from augmented reality and virtual reality apps in medical training, surgery, rehabilitation, and patient education. Alternatively, M-LLMs might be trained to analyze interactions during remote care consultations for enhanced care delivery, detect inconsistencies in patient reporting, and identify visual cues of patient discomfort or illness.

### Privacy-Preserving Analysis

**Figure 1:**
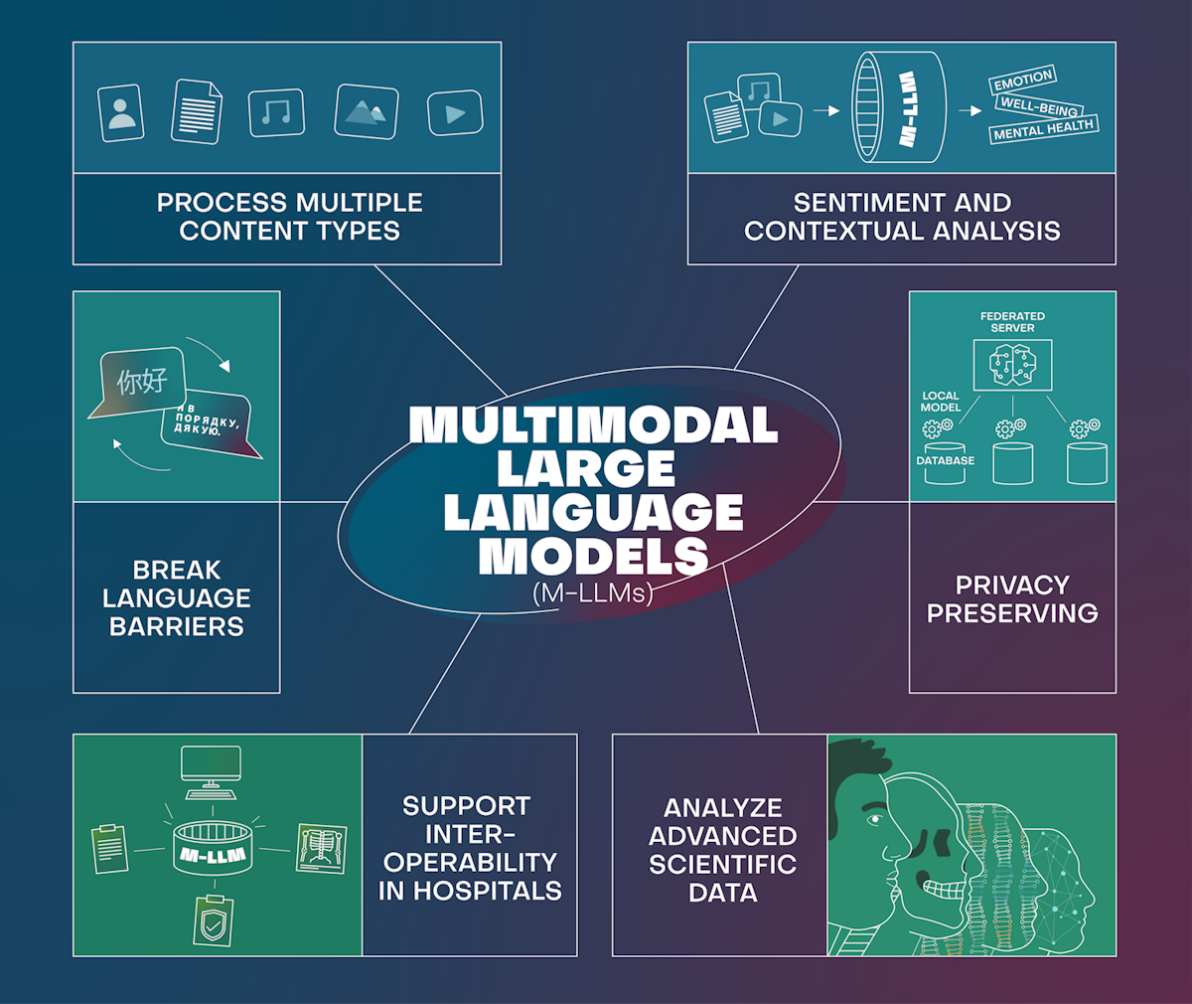
A summary of the potential high-level and long-term benefits of using multimodal large language models (M-LLMs) in health care. Examples include M-LLMs being able to (1) being able to process multiple content types (from text and video to image and sound), (2) break language barriers, (3) support interoperability in hospitals, (4) analyze advanced scientific data, (5) help preserve privacy, and (6) support sentiment and contextual analysis.

As concerns about data privacy continue to grow, future models could focus on analyzing data while preserving user privacy, implementing techniques like federated learning or differential privacy [7,8] (Figure 1).

## The Barriers and Limitations of the Vision of M-LLMs

While the potential of M-LLMs is exciting, there are substantial challenges and limitations to consider.

The most prominent question is about technical feasibility. The integration of text, image, video, sound, and other data types into one model presents significant technical challenges. LLMs currently operate on tokens, which are textual units. Extending these models to interpret and generate nontextual data such as pixels in images and frames in videos while the user would still interact with the model through text is a complex task that will require substantial advances in AI technology and computational capabilities.

Second, training an M-LLM would require large, diverse, and well-labeled data sets spanning all modalities the model is expected to handle. Such multimodal data sets are currently limited, and collecting them poses logistical, ethical, and privacy challenges.

Third, AI models trained for specific tasks (eg, analyzing medical images) have undergone rigorous testing and approval processes to ensure their reliability and safety. Integrating such task-specific models into an M-LLM is technically challenging due to differences in neural network architectures, potential data inconsistencies, and the need to preserve the integrity of each model’s specialized functionality. It would require careful validation to ensure that the M-LLM maintains the accuracy and reliability of the integrated models.

Also, there is currently a lack of regulation around AI models, especially those as complex and potentially impactful as M-LLMs. These models would need a robust regulatory framework to ensure their safe and ethical use, and creating such a framework is a complex task involving technical, legal, and ethical considerations.

Besides regulatory challenges, M-LLMs would likely handle sensitive data such as medical records or patients’ health personal records, raising substantial privacy concerns. Ensuring the confidentiality of user data, especially in areas like health care, is critical. Furthermore, privacy regulations differ globally, adding another layer of complexity to the use of M-LLMs. Also, medical professionals using M-LLMs would have to clearly state that while working with patients.

There is also a philosophical problem. Understanding how an M-LLM makes its predictions is crucial for trust and safety, especially in high-stakes areas such as health care. However, AI models are often described as “black boxes” because their decision-making processes can be difficult to interpret. Moreover, AI models can unintentionally learn and propagate biases present in their training data, and M-LLMs would be no exception.

In summary, while M-LLMs hold potential, their development and implementation pose substantial challenges that need to be carefully considered and addressed.

## What Should the Introduction of These Future Versions of M-LLMs and Their Potential Use Cases Lead to Today?

Integrating the ability to analyze documents, research papers, sound, images, and videos into LLMs could be transformative in the field of health care and medicine. However, these advancements would come with several important implications and require many stakeholders to act today.

It would be crucial, even during the current LLM revolution, to teach prompt engineering as a special skill and knowledge in medical education and training. Prompt engineering refers to the practice of designing, refining, and implementing prompts or instructions that guide the output of LLMs to help with various tasks [9].

As AI becomes more integrated into health care, it is essential that medical professionals are trained not only in how to use these tools but also in understanding their limitations. This could involve teaching prompt engineering, data science basics such as the foundational knowledge and skills related to data analysis and interpretation, and AI ethics as part of the medical curriculum. Understanding AI and data analysis could be as crucial as anatomy or biochemistry for future physicians.

Preparing the health care workforce for using M-LLMs could also help improve not only AI but specifically LLM literacy among patients, as patients also need to become more AI literate. As the use of AI by patients will likely increase with the generative AI revolution, medical professionals are expected to serve as patients’ guides in handling the use of AI and the information obtained by using it. This could involve understanding how AI is used in their care, what data are used, and the potential risks and benefits.

Regulatory bodies would need to develop robust standards and guidelines to ensure the safety, effectiveness, and ethical use of LLMs. This process includes defining what constitutes acceptable accuracy for different AI apps, ensuring transparency in AI algorithms, and protecting patient data privacy [10].

Developing and deploying LLMs in health care requires collaboration between diverse fields, including medicine, computer science, data science, ethics, and law. Interdisciplinary collaboration should be encouraged in research, education, and the development of new technologies.

As one of the key factors in the wide adoption of such a technology, evidence-based medicine must apply to LLMs too. Regardless of AI’s potential, clinical validation is key. There is a growing need to design rigorous prospective assessments through clinical trials to evaluate these models’ performance and ensure that the use of AI improves upon current standards of care without leading to harmful unintended consequences. The US Food and Drug Administration has been providing examples of this approach regarding the regulation of AI-based medical technologies [3].

## Conclusions

By presenting several futuristic scenarios and potential features of M-LLMS, we aimed at illustrating the possible path forward as M-LLMs could represent the gateway between health care professionals and using AI for medical purposes. In short, M-LLMs could facilitate the interaction with AI-based technologies.

Generative AI has been on the rise as can be seen in the number of studies and papers discussing its potential, medical associations releasing guidelines about its challenges, and even health care institutions launching collaborations with technology companies to stay competitive [11].

One example of the latter is the partnership between Google Cloud (Google LLC) and the Mayo Clinic focused on generative AI [12]. They will deploy a new HIPAA (Health Insurance Portability and Accountability Act)–compliant Google Cloud service that enables health care providers to create a search system for their data, equipped with conversational features using LLMs.

In another example, researchers from Google Research and Google DeepMind developed Med-PaLM Multimodal, an LLM that encodes and interprets biomedical data such as clinical language, medical pictures, and genetic data [13]. In that paper, authors provided a proof of concept for a generalist biomedical AI system for which, in a side-by-side ranking on 246 retrospective chest x-rays, clinicians expressed a pairwise preference over reports produced by radiologists in up to 40.50% of cases. These show the practical implications of M-LLMs materializing today.

M-LLMs have already been shown to contribute to the practice of medicine in multiple domains, from radiology (analyzing chest x-rays) to few-shot learner models adapted to the medical domain and generalist foundation models [14-16].

In summary, M-LLMs can enhance patient-physician interactions by aiding in the analysis and interpretation of medical images, videos, and text data. For instance, M-LLM’s ability to understand and analyze complex medical imagery such as skin lesions or surgical techniques can greatly assist both patients and medical professionals in initial diagnosis and skill development, respectively. Similarly, its capacity to identify potential drug-drug interactions in treatment plans can potentially prevent harmful side effects and contribute to safer patient care.

This will not only alleviate the administrative burden on health care providers but also reduce errors, leading to increased efficiency and cost savings. As a potential consequence of these, M-LLMs could finally fill in the health care HR gap [17].

Despite these potential benefits, an underlying consideration emerges. Because M-LLMs are built on tokenizing information, every content type and data set will have to be translated back into this tokenized form. From one perspective, this token-based framework could streamline interactions for physicians, allowing them to engage more intuitively with various forms of content and data. By translating complex visual or auditory information into text-based insights, M-LLMs could render intricate medical analyses more accessible and interpretable.

However, the conversion of nuanced visual or auditory data into tokens could risk a loss of information fidelity or introduce ambiguities, particularly when dealing with intricate medical imaging or subtle auditory clues. The ability to retain the integrity and richness of the original data while translating it into a text-based format might require careful consideration, calibration, and potentially the development of new tokenization strategies tailored to the medical context.

As these models become integrated into health care practices, it is essential to address ethical, technical, and legal implications, such as data privacy and the reliability of AI-generated advice, to ensure safe and responsible use.

It is important to point out, though, that despite the unprecedented potential of generative AI in the form of M-LLMs, the human touch in medicine remains irreplaceable. AI should be seen as a tool that can augment health care professionals rather than replace them. It is also important to consider the human aspects of health care—empathy, understanding, and the doctor-patient relationship—when deploying AI.

## Appendix

Multimedia Appendix 1 A summary of examples and potential cases for each content type future multimodal large language models might be able to handle.

## Abbreviations

AI: artificial intelligence
GPT: Generative Pre-trained Transformer
GPT-4: Generative Pre-trained Transformer 4
HIPAA: Health Insurance Portability and Accountability Act
LLM: large language model
M-LLM: multimodal large language model
