# Supplementary material for: The Impact of Multimodal Large Language Models on Health Care’s Future
Source: J Med Internet Res. 2023 Nov 2;25:e52865. doi: 10.2196/52865 (PMC10654899; doi:10.2196/52865)
Supplement: Multimedia Appendix 1 [file jmir_v25i1e52865_app1.docx]

**Table S1**. A summary of examples and potential cases for each content type future multimodal large language models might be able to handle.

| 1. **Text Analyses** | | | |
| --- | --- | --- | --- |
| **1.1 Administrative Tasks** | **1.2 Clinical Tasks** | **1.3 Communication and Education** | **1.4 Digital Content Creation** |
| Gathering and updating patient demographic and insurance information | Reviewing lab results | Explaining diagnosis and treatment options to patients | Developing the code for a website of a medical practice |
| Documenting patient encounters and medical histories | Developing treatment plans | Providing lifestyle recommendations (e.g., diet, exercise, stress management) for patients | Creating content online |
| Updating electronic health records (EHRs) |  | Patient interactions presenting e.g. blood pressure measurements | Creating presentations |
| Completing insurance forms and writing insurance letters |  |  | Solving and creating case studies |
| Coding medical services and procedures for billing purposes |  |  |  |
| 1. **Image analyses** | | | |
| **2.1 Documentation and Administrative Tasks** | **2.2 Clinical Diagnostics and Treatment** | **2.3 Specialty Specific Applications** | |
| Reading hand-written notes, prescriptions | Analyzing radiology images (CT and MRI scans, X-Rays) | Pathology: analyzing slides of tissue samples to identify diseases such as cancer, or to detect the presence of bacteria or viruses | |
| Making sense of photos taken of digitally written discharge notes | Analyzing photos of skin lesions | Ophthalmology: analyzing images of the retina to diagnose conditions such as diabetic retinopathy, glaucoma, or age-related macular degeneration | |
| Analyzing printed ECG results | Analyzing photos of the face of a patient for genetic conditions | Radiomics: providing insights related to prognosis and therapeutic responses for diseases like cancer | |
|  | Analyzing ultrasound images | Neurology: analyzing images of the brain to diagnose conditions like Alzheimer's disease, multiple sclerosis, or strokes | |
|  | Patient interactions presenting e.g. images of skin lesions | Telemedicine: analyzing patient-provided images in telemedicine settings, aiding healthcare professionals in making diagnoses remotely | |
|  |  | Public Health: analyzing geospatial images to predict disease outbreaks, understand health impacts of environmental changes, or monitor population health | |
|  |  | Surgical Planning and Guidance: analyzing images in real-time during surgery for enhanced precision and safety | |
| 1. **Sound analyses** | | | |
| **3.1 Disease Detection and Monitoring** | **3.2 Mental and Emotional Health** | **3.3 Rehabilitation and Assistive Technologies** | |
| Looking for vocal biomarkers (e.g. changes in voice can signal neurological disorders such as Parkinson's disease or mental health conditions) | Emotional Health Monitoring: analyzing the tone and pitch of a person's voice to detect and monitor their emotional state; to detect signs of stress, anxiety, depression, or other mental health conditions; even during telemedicine consultations | Rehabilitation: In physical and speech therapy, sound analysis could be used to monitor progress and provide feedback | |
| Cough analysis to detect/differentiate respiratory diseases |  | Assistive Technology: For individuals with speech or hearing impairments, it could help translate spoken language into sign language or transcribe spoken words into text in real time, aiding communication | |
| Heart and Lung Sound Analysis: analyzing heart and lung sounds for abnormalities, assisting in early detection of conditions like arrhythmias, heart murmurs, or lung diseases |  |  | |
| Sleep Apnea Detection: analyzing sounds produced during sleep to potentially help in diagnosing sleep disorders such as sleep apnea |  |  | |
| 1. **Video analyses** | | | |
| **4.1 Rehabilitation and Therapy** | **4.2 Surgical Assistance and Training** | **4.3 Diagnosis and Monitoring** | **4.4 Communication Aid** |
| Physical Therapy & Rehabilitation: Video analysis can be used to monitor a patient's progress in physical therapy or rehabilitation | Surgical Training and Assistance: Video analysis can aid in surgical procedures by providing real-time insights or as a tool for post-procedure review and learning | Diagnosis and Monitoring of Neurological Disorders: Video analysis could help detect and monitor neurological conditions | Speech Therapy: In speech and language therapy, video analysis could help assess a patient's speech patterns, lip movements, and facial expressions |
|  |  | Remote Patient Monitoring: AI video analysis can be used in telehealth to monitor patients' physical condition and adherence to treatment | Sign Language Recognition: For patients who communicate using sign language, video analysis could be used to accurately translate sign language to text or speech in real-time |
|  |  | Psychiatry: Video analysis could be used to assess patients for signs of mental health conditions |  |
|  |  | Fall Detection: Especially for elderly care, AI video analysis could monitor individuals and alert healthcare providers or family members if a fall is detected |  |
|  |  | Disease Progression Tracking: For chronic conditions, video analysis could help track disease progression by analyzing changes in a patient’s physical condition or behaviour over time |  |
| 1. **Document analyses** | | | |
| **5.1 Research and Education** | **5.2 Clinical Decision Support** | **5.3 Public Health and Epidemiology** | **5.4 Administration and Compliance** |
| Literature Review and Research: rapidly reviewing large volumes of medical literature to extract relevant information | Clinical Decision Support: analyzing research papers and medical guidelines to provide evidence-based recommendations to physicians | Epidemiology and Public Health: analyzing research papers and public health reports to track disease trends and predict outbreaks | Regulatory Compliance: helping healthcare institutions stay updated with regulatory changes by analyzing new guidelines, policies, and regulatory documents |
| Medical Education: used as a tool to aid learning for medical students, analyzing textbooks, papers, and teaching materials | Precision Medicine: analyzing clinical studies and genomic research papers to identify individual risk factors and suggest personalized treatment plans |  | Clinical Coding and Billing: analyzing medical records and billing documents to ensure accurate clinical coding and prevent billing errors |
| Drug Discovery: analyzing research related to drug development and clinical trials, helping to identify potential new therapies or predict drug interactions and side effects |  |  |  |
| Patient Education: analyzing medical literature to generate patient-friendly summaries or educational materials |  |  |  |
